# Social vulnerabilities among immigrants and refugees in emergencies and disasters: a systematic review

**DOI:** 10.3389/fpubh.2023.1235464

**Published:** 2024-03-07

**Authors:** Mohammad Mahdi Doust Mohammadi, Ibrahim Salmani, Hojjat Farahmandnia

**Affiliations:** ^1^Department of Health in Disaster and Emergencies, School of Public Health, Shahid Sadoughi University of Medical Sciences and Health Services, Yazd, Iran; ^2^Health in Disasters and Emergencies Research Center, Institute for Futures Studies in Health, Kerman University of Medical Sciences, Kerman, Iran

**Keywords:** social vulnerabilities, immigrants, refugees, disasters, emergencies

## Abstract

**Background:**

Due to cultural, economic, and societal factors, immigrants and refugees are pivotal groups in dealing with social vulnerability in disasters. Ignoring or inadequate attention to those groups in preparing for and responding to disasters and health emergencies could decrease the effectiveness of efforts. This article aims to identify the most basic social vulnerabilities among immigrants and refugees and provide effective solutions to alleviate or eliminate these vulnerabilities.

**Methods:**

This systematic review was performed based on the Preferred Reporting Items for Systematic Reviews and Meta-Analyses (PRISMA) guidelines. The main keywords include Social Vulnerabilities, Immigrants, Refugees, and Disasters. All articles published up to February 2023 were reviewed regardless of language and location. A total of 575 articles were extracted from SCOPUS, Web of Science, ScienceDirect, ProQuest, PubMed, EMBASE, and PsycINFO databases, and finally, 14 articles were selected for full-text analysis. The Strengthening the Reporting of Observational Studies in Epidemiology (STROBE) was used to evaluate the quality of the selected articles.

**Results:**

Fourteen articles including 4 qualitative and 10 quantitative articles were selected and analyzed in this review. The findings showed: 1. According to the consensus of the studies, the most vulnerable people who need urgent care during an epidemic due to their special conditions are immigrants and refugees; 2. In most countries, no database provides reliable, up-to-date, and accurate statistics about these people; 3. Refugees usually hesitate to express their vulnerability and receive services due to the fear of deportation; and 4. The main challenges faced by refugees are socio-economic problems such as language problems, lack of emotional and social support, and living in crowded places.

**Conclusion:**

Considering the prevalence of migration among countries, it is essential to identify the social problems and vulnerabilities of immigrants and provide effective solutions to cope with their challenges, especially during crises and emergencies.

**Systematic review registration:**

https://clinicaltrials.gov/, identifier CRD42022371345.

## Introduction

Disasters caused by terrorism have become common throughout the world. Thus, the number of people who migrate to safe areas is increasing. According to the United Nations’ International Organization for Migration (IOM), the number of international migrants grew to 281 million in 2020, indicating that 3.6% of the world’s population lived outside their country of birth that year (1). In recent years, the number of international immigrants has been increasing each year. For instance, from 2000 to 2015, Europe attracted the most international immigrants after Asia (2). The IOM describes a “migrant” as any person moving across an international border or within a country away from their usual residence (3). Migration is a social determinant adversely affecting a person’s health as the person’s health and living conditions may change before, during, and after migration (4). Migration may induce various responses in host countries, as it significantly changes social and political dimensions. Immigration has an impact on public opinion and public services, including health and medical care (5). This has led to the adoption of new laws on access to health care for immigrants, which may pose risks to public health (6).

Vulnerability is a complicated subject. The complex factors that make people vulnerable are not always immediately apparent. Vulnerability includes economic, social, cultural, political, and psychological factors that shape people’s lives and the environment in which they live. There may be various forms of vulnerability in different societies and times. The most important risk factor in extensive disasters is often the vulnerability of people. Furthermore, some social groups are more vulnerable than others. For instance, special groups, including women, children, the older adult, the disabled, immigrants, and displaced populations are often more vulnerable to the damaging effects of hazards (7). The term “social vulnerability” describes a community’s ability to withstand external stresses on human health, such as disease outbreaks or natural or man-made disasters. Social vulnerability reduction can reduce human suffering and financial loss (8). One of the most important reasons for social vulnerability is social differences or social inequality, which affects the response of societies and changes their capacity to cope with and adapt to certain disastrous situations (9).

Various factors influence social vulnerability in disasters, including lack of access to resources (information, knowledge, and technology); limited access to power and politics; social capital (social networks and communication); beliefs and customs; location; disability and physical limitations; the type and density of infrastructure; age, race, and socioeconomic status (10). In recent years, with the change in countries’ policies regarding the non-acceptance of refugees and their illegal entry into a country, the social vulnerability of these people has increased (11). COVID-19 has been of the most critical challenges in recent years leading to social vulnerability among immigrants, especially in developing countries (12). Health and social inequalities were revealed and intensified with the spread of this pandemic among immigrant and ethnic/racial groups. In addition, due to low health literacy and unawareness of the disease process, refugees are always hesitant to go to medical centers even in severe cases of illness (13).

Income growth reduces societal vulnerability, indicating that maintaining economic growth may be a key strategy for helping the world’s developing nations become more resilient and disaster-adaptive (14). According to the findings of the numerous review studies, poverty, exclusion, marginalization, and imbalances in material consumption all contribute to social vulnerability as a result of natural disasters (15).

Considering the importance of immigration, especially the problems caused by immigrants in different countries, as well as the fact that these people are forgotten during disasters, recognizing and determining the social vulnerability of this group at the time of disasters is considered a challenge. Moreover, specifying measures generally taken by other countries to solve this challenge can help managers implement standard rehabilitation plans for vulnerable immigrant and refugee groups. Therefore, this study aims to collect information from limited studies in this regard and present it in a single and focused format.

## Methods

This systematic review was conducted according to the Preferred Reporting Items for Systematic Reviews and Meta-Analyses (PRISMA) guidelines. The protocol for this systematic review was registered on Prospero (CRD42022371345).

### Search strategy

The main keywords including Social Vulnerabilities, Immigrants, Refugees, and Disasters were searched using operators AND, OR in SCOPUS, Web of Science, ScienceDirect, ProQuest, PubMed, EMBASE, and PsycINFO databases. The complete search strategy is shown in Table 1. The keywords were selected based on studies conducted in this field as well as Medical Subject Headings (MeSH). The search was done in the abstracts, keywords, and titles of the article. In addition, the reference list of published studies was also searched to find related articles. All articles published up to February 2023 were reviewed regardless of language and location.

**Table 1:** Keywords and search strategy.

"Social vulnerabilit*"	AND	Immigrant*	OR	Refugee*	AND	Disaster*
OR		OR		OR		OR
"Socia* economica* vulnerability"		Emigrant*		"Political Asylum Seeker*"		"Natural disaster*"
OR		OR		OR		OR
"soci* vulnerabilit*"		Foreigner*		"Asylum Seeker*", Political"		"Man-made disaster*"
OR		OR		OR		OR
"Vulnerabiliti*, Socia*"		Alien*		"Seeker*, Political Asylum"		crisis
OR				OR		OR
"Social Vulnerabilities"				"Political Refugee*"		hazard
OR				OR		catastrophe
"Vulnerability, Social"				"Refugee*", Political"		OR
OR				OR		tragedy
"Vulnerabilities, Social"				"Asylum Seeker*"		OR
				OR		"mass casualty Incident"
				"Seeker*, Asylum*"		OR
				OR		Emergencie*
				"Displaced Person*"		OR
				OR "Person*, Dispaced"		WAR
				OR		OR
				"Internally, Displaced Person*		conflict
				OR		OR
				"Displaced Person*, Internally"		Droughts
						OR
						Earthquakes
						OR
						Floods
						OR
						"Climate change"

* means search strategy.

### Screening process

After searching the articles in the source database, they were entered into the EndNote software. After removing duplicate and unrelated titles, abstracts, and full text of the remaining titles, the first author (IS) and the second author (MD) were screened. If discrepancies occurred between reviewers, the reasons were identified and a final decision was made based on agreement by a third reviewer (HF).

**Figure 1:**
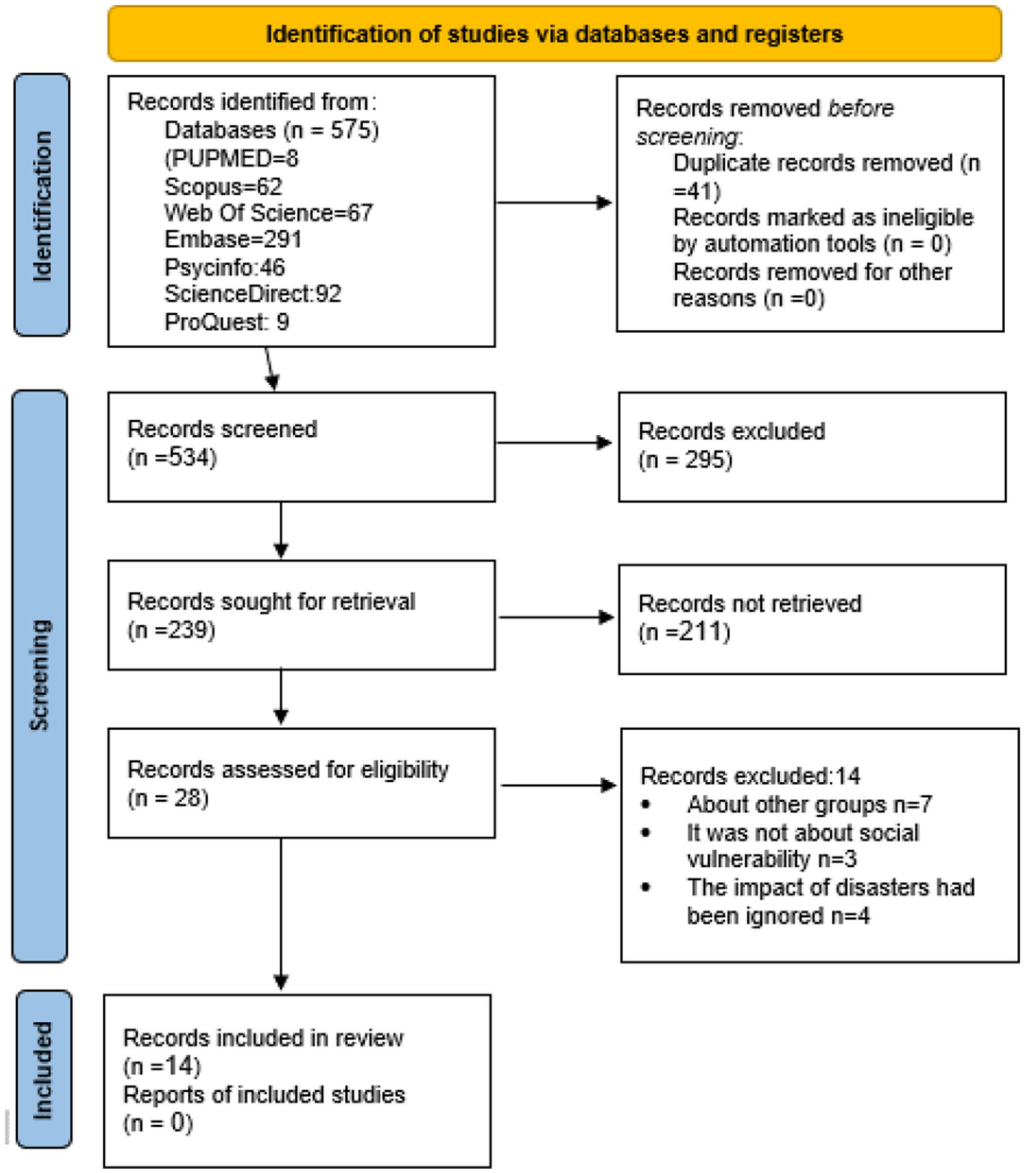
Flow diagram showing a selection of articles reviewed. Adapted with permission from Page et al. (16), licensed under CC BY 4.0.

The articles were screened by matching keywords and titles related to the social vulnerability of immigrants and refugees in disasters. In the initial search, 575 articles were obtained from different databases, from which 41 duplicate and unrelated titles were removed. The titles of the remaining 534 articles were reviewed and 239 related articles were selected. The abstracts of the 239 articles were reviewed, and 28 related articles were screened. In the next step, 14 articles were excluded because they had addressed other groups (*n* = 7) and had not focused on social vulnerability (*n* = 3) and the impact of disasters (*n* = 4). Finally, 14 articles remained as shown in Figure 1. The data of the selected articles were extracted in the pre-designed form by the first and second authors.

### Inclusion and exclusion criteria

The inclusion criteria were all original articles and reviews of qualitative and quantitative types that have been published until February 2023. Review studies, conference papers, letters to the editor, editorials, and reports were excluded.

### Quality appraisal

The Strengthening the Reporting of Observational Studies in Epidemiology (STROBE) checklist was applied for the qualification assessment of the articles (17). The checklist contains 22 questions. A “yes” answer gets 1 point and “no or not sure” gets 0 points (Table 2).

**Table 2:** Quality of the final extracted articles using strengthening the reporting of observational studies in epidemiology (STROBE).

Article	1	2	3	3	4	5	6	7	8	9	10	11	12	13	14
Title and abstract	√	√	√	√	√	√	√	√	√	√	√	√	√	√	√
Introduction															
Background/rationale	√	√	√	√	√	√	√	√	√	√	√	√	√	√	√
Objectives	√	√	√	√	√	√	–	–	√	√	√	√	√	√	√
Methods															
Study design	√	√	√	√	√	√	√	√	√	√	√	√	√	√	√
Setting	√	√	√	√	√	√	√	√	√	√	√	√	√	√	√
Participants	√	√	√	√	√	√	√	√	√	√	√	√	√	√	√
Variables	–	√	√	√	√	√	√	–	√	√	–	√	√	√	√
Data sources/measurement	√	√	√	√	√	√	√	√	√	√	√	√	√	–	√
Bias	–	√	√	–	√	–	–	–	–	–	√	–	–	√	–
Study size	√	√	√	√	√	√	√	√	√	√	√	√	√	√	√
Quantitative variables	√	√	√	√	√	√	√	–	√	√	√	√	√	–	√
Statistical methods	–	√	√	√	√	√	–	√	√	√	√	√	–	√	√
Results															
Participants	√	√	√	√	–	√	√	√	√	√	√	√	√	√	√
Descriptive data	√	√	√	√	√	√	√	√	√	√	√	√	√	√	√
Outcome data	√	√	√	√	√	–	√	√	√	√	√	√	√	√	√
Main results	√	√	√	√	√	√	√	√	√	√	√	√	√	–	√
Other analyses	√	√	√	–	√	–	√	√	√	–	√	√	√	√	√
Discussion															
Key results	√	√	√	√	√	√	√	√	√	√	√	√	√	√	√
Limitations	–	–	√	√	–	–	√	–	√	√	–	–	–	√	√
Interpretation	√	√	√	√	√	√	√	–	√	√	√	√	√	–	√
Generalizability	√	√	√	√	√	√	–	√	√	–	–	√	√	√	√
Other information															
Funding	√	–	–	–	√	√	–	–	√	–	–	–	–	√	√
STROBE grade	18	21	21	19	19	18	17	16	22	18	18	19	18	19	23

## Results

### Demographics

The number of participants in these 14 studies was 135,553 persons in total. The reason for the large number of participants in the studies is due to the use of the census method in collecting information in some articles. Details of each study and their special features including the authors, year of publication, study type, participants, problem, and solutions were reported. The studies were conducted in Asian countries (18–21), Europe (12, 22, 23), and the United States (4, 10, 20, 24–29) during disaster especially pandemic despises. After the final review of the articles, 14 main articles were selected according to the keywords and the purpose of the study.

In these articles, the main issues related to social vulnerability among immigrants and refugees were extracted, and the solutions and suggestions of the authors as well as the programs that were developed and implemented to reduce the vulnerability of this group in society were identified.

### Main results

#### Socio-cultural issues

When reviewed articles in disaster the first important problem addressed is the lack of familiarity with the local language (12, 24–26, 28, 30). Cultural changes, unawareness of the new culture, and as a result, cultural-social isolation have also been considered problems faced by immigrants (22, 24, 30). It is useful to create communication channels between the officials and immigrants to announce their problems and participate in solving the problems (10, 25). Some support centers should also be launched for immigrants regardless of the type of migration. Partnership with organizations trusted by immigrants can provide them with information about the language and cultural issues especially in climate change (18, 21, 30), racism, and xenophobia toward immigrants and refugees and help them to have adequate access to vital services such as health care (23, 27, 30). Furthermore, enough information should be provided to local people about immigrants and their problems to prevent racism, it can be said that these issues are the basis of social vulnerability in disasters for immigrants and refugees (18, 19, 23).

#### Socio-economic issues

The lack of suitable housing is also a significant problem, especially during diseases leading to an increase in the number of migrant patients and victims (27). Another important issue, especially among illegal immigrants and refugees, is expulsion from the country and the unpredictable future (23–25, 30). One of the most important solutions is to reduce tension and end civil wars (19). It is necessary to provide suitable housing and offer useful solutions for the accommodation of immigrants to prevent infectious diseases during pandemic (27). To reduce poverty in immigrant communities, it is appropriate to increase education and also increase employment opportunities (3, 26).

#### COVID-19 pandemic and immigration

More than half of the articles have addressed the Covid-19 epidemic and the vulnerability of immigrants and refugees (4, 12, 18, 22, 25, 27, 28). The most important vulnerability of this group includes livelihood problems (22, 25, 30) and housing and living problems (27) as well as vulnerability caused by limited access to vaccination facilities and medical care (20, 22). Problems related to education and social isolation can also severely affect this group. Some studies have offered solutions to reduce the vulnerability of immigrants (22, 25, 30). For instance, changing government policies can assist immigrants and provide opportunities for education and vaccination, and access to public health services.

In 2 articles, no solution has been offered for the problems faced by immigrants and refugees (Table 3). The quality of the articles varied from 7 to 22 (Table 2).

**Table 3:** Summary of articles related to the social vulnerability of immigrants and refugees.

Country	Authors	Methodology	Year	Participants	Problem	Solutions
USA	Mayfield-Johnson et all (10)	Qualitative study	2020	22	Important factors of social vulnerability Direct (*Socio-cultural issues*): racial discrimination, gender, age at migration, trauma and difficulty in understanding the language. Indirect (Socio-economic issues): Income reduction in migrants during disasters due to job loss and lack of financial support, Loss of food security.	The best solutions to reduce social vulnerability are: Understanding the ethos, culture, and vulnerability practices of the community. For increase community resilience, the Community Health Advisor (CHA) volunteer project Developing a social network for sharing information
Malaysia	Aung et al. (18)	Quantitative study	2022	283	Important factors of social vulnerability in COVID-19: Direct (Socio-cultural issues): wide gaps in access to resources and facilities necessary, Isolation, Failure to receive government helps, Lack of information and communication about pandemic disease, “Covid-19 Social Stigma” leading to violence and arrest. Indirect (Socio-economic issues): live in overcrowded homes, no access to educational opportunities, employment, and health careو Disruption of daily economic activities and loss of income, Lack of support for the disabled and children	The best solutions to reduce social vulnerability are: Raising awareness by the government prevent the dangers of violence and xenophobic behavior Providing health services and preventive measures, without discrimination Providing adequate housing to density reduction and isolation Providing support for people with disability, older people and children
Yemen	Kandeh and Kumar (19)	Qualitative study	2015	–	Important factors of social vulnerability: Direct (Socio-cultural issues): lack of education, lack of basic health services, governance is weak Indirect (Socio-economic issues): lack of proper potable water and sewage system, electricity, quality housing, poverty, limited livelihood opportunities, disputes and armed conflicts increase the social vulnerability	The best solutions to reduce social vulnerability are: The first stage of disputes, armed conflicts, and war must end. Increasing job opportunities and other livelihood opportunities. Increasing educational facilities. Access to affordable health, clean water, and electricity. Poverty reduction
USA	DeYoung et al (24)	Quantitative study	2020	326	Important factors of social vulnerability: Direct (Socio-cultural issues): lack of familiarity the language, adjusting to a new culture Indirect (Socio-economic issues): influx of refugees from war-torn, Environmental destruction	The best solutions to reduce social vulnerability are: The best solutions to reduce social vulnerability are: Intervening in the “root causes” of vulnerability, such as pervasive poverty, by relying on an appropriate source of income Addressing other essential community needs (e.g., paved roads, access to health, and education services)
USA	Sohn and Aqua (25)	Quantitative study	2022	128,528	Important factors of social vulnerability: Direct (Socio-cultural issues): structural racism and xenophobia, Distance from relatives and friends Indirect (Socio-economic issues): economic problems, loss of income and employment, marginalization, and lack of support from institutions, lack access to health care	The best solutions to reduce social vulnerability are: Removal of exclusive policies against immigrants Setting up and conducting free vaccination Identification of illegal immigrants
Chile	Blukacz et al. (4)	Quantitative study	2022	1,690	Important factors of social vulnerability: Direct (Socio-cultural issues): the lack of social support, social networks Indirect (Socio-economic issues): The problem of immigration status, living conditions, income regularity, Unemployment, reduced income	The best solutions to reduce social vulnerability are: Facilitating and simplifying immigration processes Creating institutions and support associations Not considering immigration status as a requirement to receive government money and food Guaranteeing the right to access health care regardless of immigration status
USA	Chen et al. (26)	Quantitative study	2007	113	Important factors of social vulnerability: Direct (Socio-cultural issues): social support, acculturation level, previous traumatic experiences, language barriers, lack of political power Indirect (Socio-economic issues): The problem of immigration status, living conditions, income regularity, Unemployment, reduced income, Physical health	The best solutions to reduce social vulnerability are: Social support Promoting emotional security Increasing feelings of security The support of the church Religiosity is effective in reducing psychological problems
Pakistan	Jamal-Uddin et al. (20)	Quantitative study	2017	400	Main broader conditions identified contributing to vulnerability Physical/material condition, Constitutional/organizational condition, Motivational/attitudinal condition; The illiteracy rate; poverty in society; gender, race, and regional culture	The best solutions to reduce social vulnerability are: Improving the economic situation Diversified source of income; Educating by increasing community preparedness Awareness about their vulnerable situation Response to risk Implementing disaster risk management
France	De Jesus et al. (12)	Qualitative study	2022	75	Important factors of social vulnerability: Direct (Socio-cultural issues): Disrupted official social networks, social isolation and loneliness, Concern for themselves and their close family members, social exclusion, Reliance on informal support networks Indirect (Socio-economic issues): Following public health guidelines; Food and financial insecurity; Disturbed daily and weekly routines, marginalization, and fear of eviction, Uncertain future	The best solutions to reduce social vulnerability are: Special attention from policymakers Given the migrants’ adverse living situations Sociodemographic Interventions adapted to the linguistic, cultural Civil society organizations
China	Xu and Takahashi (21)	Quantitative study	2021	100	Important factors of social vulnerability: Direct (Socio-cultural issues): Separation from the indigenous people, the problems of social and government institutions, unsustainable use of resources, leading to instability and insecurity of household livelihoods, Indirect (Socio-economic issues): Communication between local authorities and immigrants is always top-down, the environmental and economic risks of fishing are transferred to the immigrants	The best solutions to reduce social vulnerability are: Developing more effective policies to protect these groups Eliminating prejudice and integrating new immigrants into society Reforming the immigrant registration system Establishing social organizations that accommodate more immigrants Reducing dependence on marine resources for livelihood
USA	McConnell (27)	Quantitative study	2017	3,000	Important factors of social vulnerability: Direct (Socio-cultural issues): communicating, coordinating among households, Language problems, The problem of education, especially in children Indirect (Socio-economic issues): Legal asylum, Suitable housing, Cost reduction and poverty, Uncertainty of work and employment, Uncertain and temporary contracts, Quarantine and curfew and reduction of income, Lack of resources for treatment, Lack of vaccination	The best solutions to reduce social vulnerability are: Provision of suitable housing Considering these people in informing and also informing them about the disease The activation of social support and recovery measures Quick response in the first stage of the crisis and to respond to the primary and secondary needs of nutrition, shelter, safety, and clothing. The promotion of practical and virtual education at the community level
USA	Kim and Bostwick (28)	Quantitative study	2020	31,508	Important factors of social vulnerability: Direct (Socio-cultural issues): The persistence of social inequalities, including poverty, racial discrimination, and spatial exclusion, language problem Indirect (Socio-economic issues): health inequalities; Racial/ethnic, social, and economic disparities, no plan to have clean water, healthy food, and proper transportation during disasters. Unsanitary place	The best solutions to reduce social vulnerability are: Health policies must address broader structural inequalities. Public health can only be protected by reducing the social vulnerability of all communities.
USA	Flores et al. (29)	Quantitative study	2020	988	Important factors of social vulnerability: Direct (Socio-cultural issues): The language problem, Receiving the least amount of government aid and health assistance during disasters. Indirect (Socio-economic issues): Not having clean water, healthy food, and proper transportation during disasters; Unsanitary place and destruction of houses during calamities.	No recommended by paper
New Zealand	Uekusa and Matthewman (23)	Qualitative study	2017	28	Important factors of social vulnerability: Direct (Socio-cultural issues): social inequality daily, oppression and discrimination Indirect (Socio-economic issues): Not having clean water, healthy food, and proper transportation during disasters; unsanitary place and destruction of houses during calamities	The best solutions to reduce social vulnerability are: Developing durable social networks to depend each other Prior experiences Practical knowledge Cultural values and attitudes of how to support each other Cultural capital

## Discussion

The first problem faced by immigrants is not knowing the language spoken in the host community (12, 24–26, 28, 30). Thus, refugees have problems understanding the warnings, so they cannot take effective and useful measures to reduce the effects of disasters. They have difficulties asking for help and communicating with the natives of the region.

Being away from relatives and friends and having difficulty adapting to a new culture may create mental health disorders such as anxiety, depression, anger, and guilt in immigrants (23–25, 30). Thus, providing social support can improve immigrants’ health status (26). Hurricane Katrina showed that social network has a vital impact and is more effective than other factors in the management of disasters (28, 30). Usually, communication between local authorities and immigrants is always top-down. Immigrants are considered to be temporary residents who lack membership in the society, hence the authorities do not feel responsible for the catastrophic problems of immigrants. This approach reduces the resilience of these people against socioeconomic and environmental shocks (12, 21). Thus, there is a need for strengthening the social and communication networks of immigrants through communicating with charitable centers and organizations trusted by immigrants to provide health and care services for this group of people and disseminating information needed by immigrants, especially during disasters, based on the language and culture of this group of people (10, 25).

Structural racism and xenophobia toward immigrants and refugees make adequate access to vital services such as health care problematic. Kim et al. examined the available information about native people and the areas where immigrants live and compared it with the death rate caused by the COVID-19 pandemic. They concluded that most of the human casualties happen in regions where people of African descent live. Structural factors, such as poverty, segregation, and discrimination, affect the ability to recover from disasters. These inequalities destroy society’s capacity to deal with disasters and cause an increase in deaths (29). On the other hand, during reconstruction, immigrants and workers from other countries suffer the most. Working with low wages and hard and harmful work without the right to protest and threat of being introduced to the immigration office in case of requesting higher salary and other benefits or leaving work increase the vulnerability of immigrants (27). Governments should prevent the risk of violence and xenophobic behavior toward refugees by increasing public awareness (18). Understanding the vulnerable community’s ethics, culture, and lifestyle can contribute to developing interventions and creating a social network based on the vulnerable population’s spirit, personality, and cultural structures (10). Social support may neutralize traumatic experiences by creating a meaningful attachment, promoting emotional security, and increasing the sense of security. The support of the church and the assistance provided is useful in reducing the stress and problems of immigrant people. On the other hand, religiosity is effective in reducing the burden of psychological problems (26, 30).

Some studies have addressed the legality or illegality of immigration (4, 30). Lack of residence and fear of deportation causes some immigrants to refuse to apply for services, especially healthcare services (24). The most important problems faced by illegal immigrants are the exclusion of education, lack of primary health services, lack of water, electricity, and sanitary sewer system, vagrancy, lack of quality housing, poverty, and limited livelihood opportunities (24, 27, 28). Creating effective policies to protect these non-represented groups, actively promoting the integration of new immigrants with the local community, reforming the civil registration system and identifying illegal immigrants, and eliminating social prejudice are vital to reducing the vulnerability of immigrants (18, 21, 30). Registering employers’ violations can increase the trust and adaptation of immigrant communities (30). Furthermore, some laws need to be amended to allow illegal immigrants to use health services without fear of deportation. In addition, immigrants and refugees need to be assured that people who are volunteered to help them in disasters have no connection with immigration organizations (12).

Significant loss of income is a concern following a disaster in an area where immigrants and refugees live (19). In the reconstruction and recovery phase, poor people experience more vulnerability (26). After Hurricane Katrina, in addition to the financial problems caused by the storm, job loss and reduced employment opportunities made most of the damage to immigrants (10, 20, 26). Poverty alleviation programs should be developed and implemented to increase job and other livelihood opportunities (18, 19, 23). Strengthening the adaptability of immigrants by relying on a suitable source of income and addressing their essential needs (such as asphalt roads, access to clean water, health services, and educational services) are beneficial for this group of people (24). Activating social and health support measures for a quick response in the first stage of the crisis and responding to the primary and secondary needs for nutrition, shelter, safety, and clothing are essential for immigrants (22).

Having suitable housing is one of the critical issues in reducing the social vulnerability of immigrants, especially at the time of infectious and epidemic diseases. Accordingly, McConnell concluded that locating, communicating, and coordinating among immigrant families before, during, and after disasters is complicated to the extent that even the additional challenges of poverty, language, and barriers to citizenship and legal status can be ignored (27).

### COVID-19 pandemic in immigrants and refugees

During the COVID-19 pandemic, due to the lack of organization among immigrants and refugees, the spread of disease and deaths were the highest in this group. Thus, immigrants have a large share of problems and vulnerabilities (4, 12, 18, 22, 25, 27, 28). Pandemics have been found to disproportionately affect the world’s most vulnerable groups, such as refugees and displaced persons (4, 25). Many of them are domestic workers who live without a regular contract, residence permit, housing, salary, and little savings. In fact, during the quarantine, the movement restriction measures prevent many migrant workers from moving and looking for job opportunities. The COVID-19 pandemic has also led to the closure of many manufacturing activities in which migrants are employed, such as hotels, bars, and restaurants. Examining and screening immigrants for COVID-19 is not easily possible. As they live in overcrowded homes and lack access to educational, employment, and healthcare opportunities, their risk of infection is high (22, 25, 30). The vulnerability of migrant children in the COVID-19 pandemic increases due to the lack of resources for treatment and vaccination. Besides, immigrant children have more educational problems during the COVID-19 pandemic due to language problems and the use of virtual education and digital educational aids. Moreover, immigrants do not often receive government aid (28). Furthermore, restricted access to educational services also increases social vulnerability in immigrants (20, 22).

Building trust in health services and non-discriminatory preventive measures will help support at-risk individuals and host populations (4, 30). Some actions that are effective in reducing the vulnerability of immigrants include removing exclusive policies against immigrants, conducting free vaccinations, identifying illegal immigrants and facilitating the immigration process, monetary and food assistance from the government, and access to healthcare regardless of immigration status (18, 28).

## Limitations

There were some limitations in this study. First, this study did not review unpublished studies. However, it included most of the recent studies. Second, there could be an element of reporting bias due to the heterogeneity of measurement tools used to assess outcomes by different authors.

## Conclusion

The social vulnerability of the immigrant minority in disasters is an important and undeniable issue, which if neglected affects all members of a community, especially during pandemics such as the COVID-19 outbreak. Social vulnerability varies depending on the type of laws and related problems and issues in different countries. Inequality and injustice are among the most important indicators that lead to social vulnerability as evident in the studies. These problems root in the culture and beliefs of the society and if neglected, they lead to the exclusion of immigrants and refugees.

Finding effective solutions to help immigrants and refugees can help reduce their vulnerability. Given the growing trend of immigration and asylum-seeking, countries should establish effective plans and laws for this vulnerable group to help them during disasters and reduce the problems faced by them. The use of government and NGO facilities to communicate with immigrants and refugees regardless of the type of migration and the country of origin can lead to the recognition of their needs and deficiencies. Moreover, creating a social network between immigrants and residents of the host community can contribute to reducing the vulnerability of this minority.

Considering the above discussion and the importance of identifying the social vulnerability and the underlying factors of this type of vulnerability and, on the other hand, eliminating the underlying factors and the conflicts between different countries, it is suggested: a joint action plan be developed by the immigration-receiving countries to reduce and compensate for this type of vulnerability, especially during disaster.

It should be noted that migrants and refugees are not temporary persons; they are natives of their country of origin and need to be planned and taken into account in times of disaster.

## Data availability statement

The raw data supporting the conclusions of this article will be made available by the authors, without undue reservation.

## Ethics statement

A review study with Reg. No. 402000585 and ethical code number IR.KMU.REC.1402.436 was approved by the ethical committee of Kerman University of Medical Sciences. All methods were performed in accordance with the relevant guidelines and regulations.

## Author contributions

IS conceived the concept and design of the study, supervised the study, and critically reviewed the manuscript. HF, AT and AF conducted the survey. MD was involved in data analysis and manuscript writing. All authors read and reviewed the final manuscript.

## References

[1] Natarajan A. Moslimani M. Lopez M.H., Key facts about recent trends in global migration. Pew Research Center (2022). Available at: https://www.pewresearch.org/

[2] United Nations Department of Economic and Social Affairs. International migration report 2015 United Nations (2015).

[3] Filčák R. Migration to contaminated sites: migrants' settlements in central and eastern Europe built in places with high environmental and social vulnerability In: Jäger J. (eds). Environment, forced migration and social vulnerability (2010). Berlin, Heidelberg: Springer.

[4] Blukacz A Cabieses B Mezones-Holguín E Cardona Arias JM. Healthcare and social needs of international migrants during the COVID-19 pandemic in Latin America: analysis of the Chilean case. Glob Health Promot. (2022) 29:119–28. doi: 10.1177/17579759211067562, PMID: 35311402 PMC9607989

[5] Adepoju OE Han D Chae M Smith KL Gilbert L Choudhury S . Health disparities and climate change: the intersection of three disaster events on vulnerable communities in Houston, Texas. Int J Environ Res Public Health. (2022) 19:35. doi: 10.3390/ijerph19010035 PMC8751109 35010293

[6] Sarría-Santamera A Hijas-Gómez AI Carmona R Gimeno-Feliú LA. A systematic review of the use of health services by immigrants and native populations. Public Health Rev. (2016) 37:1–29. doi: 10.1186/s40985-016-0042-3 29450069 PMC5810113

[7] Scott M. Finding agency in adversity: applying the refugee convention in the context of disasters and climate change. Refug Surv Q. (2016) 35:26–57. doi: 10.1093/rsq/hdw018

[8] Yan J. Putting people first: practices, challenges and innovations in characterizing and mapping social groups. in Proceedings from the 2016 UR Forum. (2016). Available at: https://understandrisk.org/wp-content/uploads/Intro-to-session.pdf

[9] Nor Diana MI Muhamad N Taha MR Osman A Alam MM. Social vulnerability assessment for landslide hazards in Malaysia: a systematic review study. Land. (2021) 10:315. doi: 10.3390/land10030315

[10] Mayfield-Johnson S Fastring D Le D Nguyen J. Addressing the social vulnerability of Mississippi gulf coast vietnamese community through the development of community health advisors. Sustainability. (2020) 12:3892. doi: 10.3390/su12093892 32714606 PMC7380657

[11] Black R. Livelihoods under stress: a case study of refugee vulnerability in Greece. J Refug Stud. (1994) 7:360–77. doi: 10.1093/jrs/7.4.360

[12] De Jesus M Moumni Z Sougui ZH Biswas N Kubicz R Pourtau L. “Living in confinement, stopped in time”: migrant social vulnerability, coping and health during the COVID-19 pandemic lockdown in France. Int J Environ Res Public Health. (2022) 19:10084. doi: 10.3390/ijerph191610084 36011730 PMC9408687

[13] Salmani I Seddighi H Nikfard M. Access to health care services for afghan refugees in Iran in the COVID-19 pandemic. Disaster Med Public Health Prep. (2020) 14:e13–4. doi: 10.1017/dmp.2020.240, PMID: 32660673 PMC7426587

[14] Ward PS Shively GE. Disaster risk, social vulnerability, and economic development. Disasters. (2017) 41:324–51. doi: 10.1111/disa.12199 27174613

[15] Singh SR Eghdami MR Singh S. The concept of social vulnerability: a review from disasters perspectives. Int J Interdiscipl Multidiscipl Stud. (2014) 1:71–82.

[16] Page MJ McKenzie JE Bossuyt PM Boutron I Hoffmann TC Mulrow CD . The PRISMA 2020 statement: an updated guideline for reporting systematic reviews. BMJ. (2021) 372:n71. doi: 10.1136/bmj.n71 33782057 PMC8005924

[17] Ghaferi AA Schwartz TA Pawlik TM. STROBE reporting guidelines for observational studies. JAMA Surg. (2021) 156:577–8. doi: 10.1001/jamasurg.2021.0528, PMID: 33825815

[18] Aung TS Fischer TB Wang Y. Conceptualization of health and social vulnerability of marginalized populations during Covid-19 using quantitative scoring approach. J Immigr Refug Stud. (2022) 20:1–16. doi: 10.1080/15562948.2021.1882023

[19] Kandeh J Kumar L. Developing a relative ranking of social vulnerability of governorates of Yemen to humanitarian crisis. ISPRS Int J Geo Inf. (2015) 4:1913–35. doi: 10.3390/ijgi4041913

[20] Jamal-Uddin SA Murtaza G Faiz S. Identifying factors of vulnerabilities to natural disasters: a case of Malgagai refugee village Killasaifullah, Balochistan. Bi-Annual Research Journal “BALOCHISTAN REVIEW” (2017). Balochistan Study Centre, University of Balochistan, Quetta Pakistan: University of Balochistan VOL. XXXVII.

[21] Xu J Takahashi M. Progressing vulnerability of the immigrants in an urbanizing village in coastal China. Environ Dev Sustainab. (2021) 23:8012–26. doi: 10.1007/s10668-020-00914-8

[22] Ferrari M. Resilience, Vulnerability And Inequality In Immigrant Children During The Covid 19 Pandemic. In Proceedings of The 4th International Academic Conference on Research in Social Sciences 2022 (2021) (Vol. 4) Diamond Scientific Publishing. 54–77.

[23] Uekusa S Matthewman S. Vulnerable and resilient? Immigrants and refugees in the 2010–2011 Canterbury and Tohoku disasters. Int J Disast Risk Reduct. (2017) 22:355–61. doi: 10.1016/j.ijdrr.2017.02.006

[24] DeYoung SE Lewis DC Seponski DM Augustine DA Phal M. Disaster preparedness and well-being among Cambodian- and Laotian-Americans. Disaster Prev Manag. (2020) 29:425–43. doi: 10.1108/DPM-01-2019-0034

[25] Sohn H Aqua JK. Geographic variation in COVID-19 vulnerability by legal immigration status in California: a prepandemic cross-sectional study. BMJ Open. (2022) 12:e054331. doi: 10.1136/bmjopen-2021-054331, PMID: 35613755 PMC9130646

[26] Chen AC Keith VM Leong KJ Airriess C Li W Chung K-Y . Hurricane Katrina: prior trauma, poverty and health among Vietnamese-American survivors. Int Nurs Rev. (2007) 54:324–31. doi: 10.1111/j.1466-7657.2007.00597.x, PMID: 17958660

[27] McConnell ED. Rented, crowded, and unaffordable? Social vulnerabilities and the accumulation of precarious housing conditions in Los Angeles. Hous Policy Debate. (2017) 27:60–79. doi: 10.1080/10511482.2016.1164738

[28] Kim SJ Bostwick W. Social vulnerability and racial inequality in COVID-19 deaths in Chicago. Health Educ Behav. (2020) 47:509–13. doi: 10.1177/1090198120929677 32436405 PMC8183499

[29] Flores AB Collins TW Grineski SE Chakraborty J. Social vulnerability to hurricane Harvey: unmet needs and adverse event experiences in greater Houston, Texas. Int J Disast Risk Reduct. (2020) 46:101521. doi: 10.1016/j.ijdrr.2020.101521

[30] Fussell E Delp L Riley K Chávez S Valenzuela A Jr. Implications of social and legal status on immigrants' health in disaster zones. Am J Public Health. (2018) 108:1617–20. doi: 10.2105/AJPH.2018.304554, PMID: 30359114 PMC6236743

